# Barrett's oesophagus: A qualitative study of patient burden, care delivery experience and follow‐up needs

**DOI:** 10.1111/hex.12817

**Published:** 2018-11-14

**Authors:** James Britton, Shaheen Hamdy, John McLaughlin, Maria Horne, Yeng Ang

**Affiliations:** ^1^ Division of Diabetes, Endocrinology and Gastroenterology School of Medical Sciences Faculty of Biology, Medicine and Health The University of Manchester and Manchester Academic Health Sciences Centre Manchester UK; ^2^ Wrightington, Wigan and Leigh NHS Trust Wigan UK; ^3^ Salford Royal NHS Foundation Trust Salford UK; ^4^ Faculty of Medicine and Health Leeds University School of Healthcare University of Leeds Leeds UK

**Keywords:** Barrett's oesophagus, dedicated service, delivery of health care, interview, needs, health services, oesophageal cancer, patient perspective, quality of health care, quality of life

## Abstract

**Background:**

Barrett's oesophagus (BO), a precursor to oesophageal adenocarcinoma, requires long‐term endoscopic surveillance. The rising incidence of this chronic disease has implications for service provision and patient burden. Few studies have explored BO patients’ personal burden, care delivery experience and participation in health‐care delivery decisions.

**Objective:**

To identify and explore factors impacting BO patients’ health‐related quality of life, follow‐up needs and views on new models of follow‐up care.

**Design:**

An exploratory qualitative approach was adopted using semi‐structured, in‐depth, one‐to‐one interviews, audio‐recorded and transcribed verbatim. Patients undergoing BO surveillance, at a single NHS hospital, were recruited using purposive sampling with the aim of achieving maximum variation. Data were analysed using framework analysis approach, supported by NVivo Pro 11.

**Results:**

Data saturation occurred after 20 participant interviews. Ten subthemes and three main themes emerged from the analysis: (a) burden of disease—symptom control, worry of oesophageal cancer and surveillance endoscopy; (b) follow‐up experiences—follow‐up care, at this NHS hospital, was found to be inconsistent and often inadequate to meet patients’ needs, in particular a lack of disease‐specific information; and (c) follow‐up needs—participants sought enhanced communication, organization and structure of care. They highly valued face‐to‐face interaction with a specialist, and the concept of direct secondary care access in‐between endoscopies was reassuring to participants.

**Conclusions:**

This qualitative research provides an in‐depth account of the patients’ perspective of BO, the effectiveness of follow‐up care and patient opinion on new follow‐up systems.

## 1 INTRODUCTION

In contrast to many other cancers in the Western world, the incidence of oesophageal adenocarcinoma (OAC) has increased over the last three decades^1^, ^2^, ^3^ with no significant change in survival over the last 10 years.^4^ In an attempt to address this imbalance, Barrett's oesophagus (BO) has been identified as a key opportunity to intervene and prevent OAC. With clearer referral guidelines^5^ and national public health campaigns (Public Health England “be clear on cancer”),^6^ the diagnosis of this precursor for OAC will continue to increase.^7^ Without reliable individual risk stratification, the majority of patients with BO undergo long‐term endoscopic surveillance, which has implications for future health‐care provision and lifelong patient burden. Few studies, predominantly quantitative in design, have demonstrated significant reductions in BO patients’ health‐related quality‐of‐life (HRQOL) scores. However, many of these are now outdated, lack generalizability and have used measurement tools not specific to BO.^8^ Only in recent years have international guidelines, the British Society of Gastroenterology (BSG) and American College of Gastroenterology, advised consultation and counselling of newly diagnosed patients prior to surveillance enrolment.^5^, ^9^ Historically, BO patients are likely to have received inconsistent care from poorly informed or even disengaged physicians.^10^, ^11^ The effects of historic follow‐up and current care pathways on patients remain unknown.

Traditionally, the providers of new health‐care developments have controlled their design and implementation. This archaic “doctor knows best” attitude to health‐care delivery and research has begun to change in the NHS over recent years with a keener focus on patient‐centred, effective and safe clinical care.^12^, ^13^, ^14^, ^15^, ^16^ One area where user involvement appears to have its greatest influence is when drawing upon patients’ experiences, particularly in chronic disease settings. Previous engagement with patients to identify and address their follow‐up needs has dramatically changed the landscape of care in some chronic diseases. Most notably, within gastroenterology, have been the developments in inflammatory bowel disease (IBD) care. In 1991^17^ Probert et al conducted a questionnaire survey regarding disease counselling preferences in 59 patients with IBD. This landmark paper identified a significant number (60%) who required further information regarding their condition. They also found that many patients would be happy with a trained nurse consultation and identified a need for more rapid access to services. Since then, the role of the specialist IBD nurse has evolved and has been proven to reduce admissions, emergency attendances and outpatient appointments leading to large cost savings.^18^ These improvements likely reflect enhancements in professional‐patient relationships, patient disease‐specific knowledge, self‐care and medication compliance. These endpoints, however, are somewhat harder to measure. More recent research in IBD follow‐up care found that patients desire more active involvement in their care and are keen to explore more novel follow‐up alternatives, for example virtual clinics.^19^

Although the disease profiles, patient demographics and treatments may differ dramatically between chronic diseases, there are valuable commonalities to draw from these patient involvement strategies and service improvements. In particular, these include the processes used to involve patients and seek alternative or enhanced ways to educate, follow up and communicate with patients.

### 1.1 Aims

- To identify and explore factors impacting BO patients’ HRQOL.
- To identify and explore the follow‐up needs of BO patients.
- To explore patients’ perceptions and attitudes to new models of follow‐up care.

## 2 METHODS

This exploratory qualitative research forms part of a concurrent mixed‐methods study, using both qualitative and quantitative data collection tools, to explore the impact of BO on patients’ HRQOL,^20^ their experiences of follow‐up care and attitudes towards service developments in line with the preliminary research needed when developing complex interventions.^21^ This qualitative approach attempts to understand the social phenomena in natural circumstances, with an emphasis on exploring meanings and views of participants.^22^ The study design incorporates the consolidated criteria for reporting qualitative research guidelines^23^ (see Appendix S1 for further details).

### 2.1 Ethical considerations

Prior ethical approval for this study was obtained from the Health Research Authority Yorkshire and Humber ethics committee (REC reference number 16/YH/0035).

### 2.2 Participants and setting

Individuals with BO, enrolled in surveillance at a single general NHS hospital, were targeted because they were readily accessible within the constraints of the study team geography. Participants were purposively^24^ recruited with the aim of achieving maximum variation in terms of disease duration, age and gender even though this is a male‐predominant disease. Recruitment continued until a point where data saturation was reached, that is where no new themes emerged from additional interviewees^25^; however, the authors recognize this remains a contested concept,^26^, ^27^ based on the researcher's subjectivity of what they are hearing.^28^ Participants were recruited face to face at their surveillance endoscopy, via telephone or postal invite. There was no prior contact between researchers and participants before recruitment.

### 2.3 Data collection

Semi‐structured, in‐depth, one‐to‐one interviews were undertaken by JB (average time of 40 minutes, range 21‐76 minutes). The status of the interviewer (postgraduate research doctor) was made aware to all participants. An interview topic guide was developed from a prior literature review^8^ and expert opinion (please see Appendix S1). Interviews focused on the impact of surveillance, physical and psychological symptoms, experiences of follow‐up care, follow‐up needs and new models of follow‐up care. New models of care included a dedicated BO service and patient‐initiated consultation by means of telephone or virtual clinic. All interviews were conducted in a private seminar room to provide a non‐clinical atmosphere. Interviews were audio‐recorded, transcribed verbatim and anonymized prior to analysis. Participant's demographics and disease‐specific information were also collected from their medical notes and endoscopy reports. Field notes were taken at the time of each interview. These written recordings captured important verbal and non‐verbal information which can be overlooked once the content is transcribed. This is an important step to keep the context of the interview.

## 3 DATA ANALYSIS

**Figure 1:**
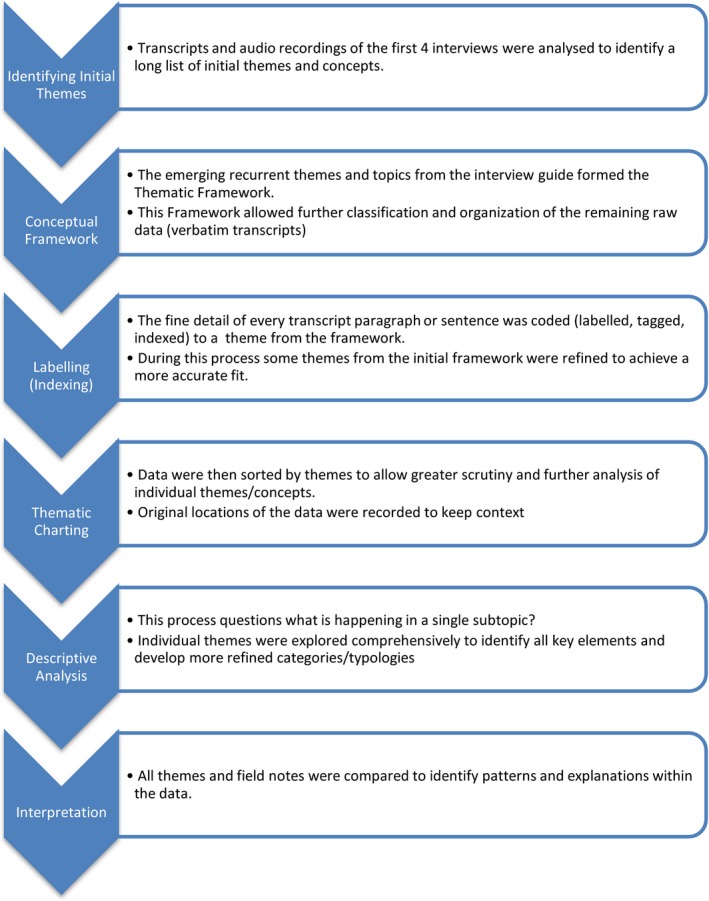
Framework analysis

A thematic analysis was conducted on all data, using a framework approach^29^ supported by NVivo Pro 11 (QSR International (UK) Limited, Cheshire UK). The key steps are outlined in Figure 1. This widely used approach^30^ allows rigorous analysis without losing transparency or site of the initial raw data. Initial emerging themes were identified from the first four interviews. These themes, alongside topics raised from the interview guide, formed the conceptual framework (Table 1). This framework was then applied manually to the raw data in a process called indexing. Field notes were linked to the content with clear associations between themes recorded for later use in descriptive analysis. The fully indexed raw data were then displayed in thematic charts allowing greater focus and distillation of the detail in each subtheme (see Appendix S1). Each column of the thematic chart was then subjected to descriptive analysis and further interpretation of the data to recognize patterns and explanations.

**Table 1:** Conceptual framework

Initial main themes	Initial categories	Contributing participants (*n*/20)	Verbatim quotes
1. Controlling symptoms	1.1 Impact of medication on symptoms	18	40
	1.2 Changes to lifestyle	20	68
	1.3 Managing symptom flare‐ups	19	40
	1.4 Attitudes/concerns regarding medication	19	31
2. Disease impact	2.1 Physical symptom impact	18	59
	2.2 Associated worries/anxieties	20	106
	2.3 Surveillance endoscopy impact	19	65
3. Disease‐specific knowledge	3.1 Disease‐specific knowledge and health beliefs	20	96
	3.2 Knowledge gaps	16	68
	3.4 Information sources	19	78
4. Follow‐up experiences	4.1 Experiences with secondary care at time of diagnosis	20	71
	4.2 Experiences of surveillance endoscopy	19	81
	4.3 Experiences with primary care (GP)	19	50
	4.4 Value of surveillance endoscopy to them	19	62
5. Follow‐up needs	5.1 Unmet needs	18	62
	5.2 Value of seeing an expert	12	31
	5.3 Other ideas offered	14	37
6. Attitudes to new models of follow‐up care	6.1 Dedicated Barrett's oesophagus service	20	77
	6.2 Patient‐initiated telephone consultation	20	78
	6.3 Patient‐initiated online consultation (“virtual clinic”)	18	39

### 3.1 Rigour

The following steps were taken to ensure rigour. Firstly, none of the participants had prior clinical contact with the researchers. The topic guide was reviewed by all researchers to ensure appropriateness of the content. Field notes were taken during each interview to ensure grounding of the content during analysis. Finally, two initial verbatim transcripts were analysed by two different researchers (JB and MH, a qualitative research specialist with a clinical background in nursing) to confirm the data were within the remit of the study and the initial emerging themes identified were consistent and fit the data captured. Preliminary findings were discussed between JB, MH and YA who agreed upon the relevance of the data and credibility of the analysis. Consensus on themes was reached through discussion.

## 4 RESULTS

**Figure 2:**
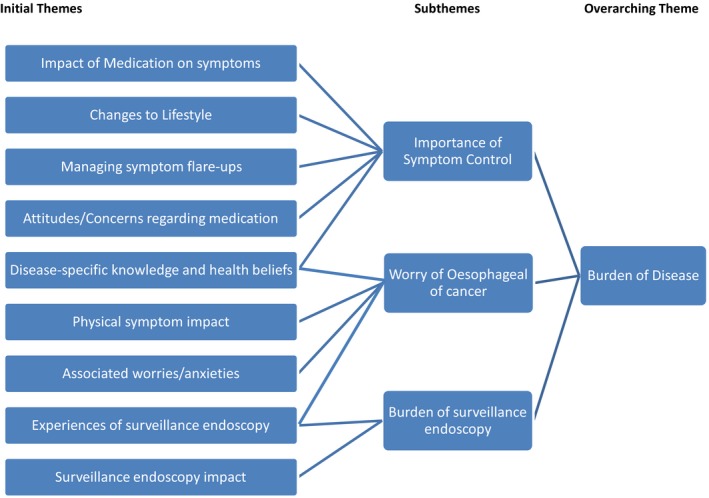
Developing an overarching theme

Data saturation, the point where no new information emerged from the data,^31^ occurred after 20 participant interviews, the demographics of which are displayed in Table 2. In total, this process generated three overarching themes and 10 subthemes (Figure 2). Considering the aims of the study, the results will be discussed under the three main themes: (a) burden of disease, (b) follow‐up experiences and (c) follow‐up needs. Information describing each theme is given and supplemented with original verbatim quotes (Table 3).

**Table 2:** Participant demographics and characteristics

Participant	Age (median = 63 y, range = 42‐77 y)	Gender	Disease duration (median = 5.8 y, range = 1‐15 y)	Prague classification (median = C3.6M5, range = C0‐10, M2‐10)	Comorbidities
A	56	M	4 y 7 mo	C2M4	Hypertension
B	71	F	2 y	C2M4	Asthma, coeliac disease, osteoporosis
C	69	M	10 y	C10M10	Hyperlipidaemia
D	42	M	4 y	C0M5	None
E	65	M	1 y 8 mo	C2M3	High cholesterol, hypertension
F	66	M	8 y	C2M3	Pulmonary fibrosis
G	58	M	7 y 1 mo	C2M4	Hypertension, musculoskeletal pain
H	62	M	2 y 2 mo	C4M6	None
I	77	M	1 y 5 mo	C2M4	Hypertension
J	46	M	4 y 6 mo	C0M2	None
K	61	F	8 y 2 mo	C1M2	Previous thyroid cancer, hypertension
L	70	M	2 y 4 mo	C6M7	Ischaemic heart disease, abdominal aortic aneurysm
M	50	M	4 y	C2M2	None
N	61	M	4 y	C9M10	None
O	76	M	6 y 6 mo	C6M6	None
P	66	M	1 y 9 mo	C2M4	None
Q	76	F	13 y 3 mo	C8M8	Rheumatoid arthritis, hypertension
R	58	F	11 y 2 mo	C8M8	Depression, osteoarthritis, previous joint replacement, previous gastric bypass
S	63	F	3 y	C4M5	Osteoarthritis
T	65	M	15 y 10 mo	C0M3	Hypertension

M, male; F, female; CnMn, circumferential and maximum BO measurement.

**Table 3:** Example verbatim quotes to supplement result sections

Result section	Verbatim quote (age, gender)	Participant ID
4.11	“It was a new lease of life for me because I wasn't having the horrible symptoms because of the tablets. I was very pleased with the tablets and I still am.” (56yr, male)	A
	“I can take my medication and not change my diet but every so often you get a really bad, severe, like burning in my throat and back pain and it feels like someone's put an axe in your back. I might be in a circle with a few friends and suddenly you have to disappear, you have to make apologies for leaving because of the pain”. (42yr, male)	D
4.12	“It's just that a lot of close people have died recently of cancer, so it's gets you thinking doesn't it. I've got a young family at home, so yeah, it's a massive thing. Every time I get symptoms I start worrying. And obviously you don't want them symptoms, you just want to live a nice healthy life.” (42yr, male)	D
	“I think I'm coming here every two years to get it checked and if there is any problem it'll be found straightaway, and that's always at the back of my mind, and that stops me from worrying about it. I know I've got this problem but it's controllable. And I don't feel of any risk of anything. I don't know if that is wrong but that's how I feel.” (69yr, male)	C
4.13	“It's terrible. It affects me for weeks before and not just on that day. Just the thought of what's going to happen. And it was an awful, awful sensation. And then it went on and on. They weren't talking to me, which is very, very important. You can't reply to them but nevertheless you want something, you know, ‘everything's fine, we're halfway through now, it won't be long now’, something like that would make a lot of a difference.” (76yr, male)	O
4.21	“When I came in and I sat down in the waiting room before I went in for my camera, the nurse told me I've got Barrett's. So, it must have been found at an earlier date and I was never informed that I'd got it.” (65yr, male)	E
	“I know time is of the essence sometimes, you know…It was sort of coming off the production line type of thing. I didn't think it was informative enough. I mean, when somebody hits you with like two different things as well, you know, Barrett's and a hiatus hernia, it said it was 2 to 3 cm. Now, that seems big to me and I didn't know what to do about it really.” (66yr, male)	P
4.22	“Your GP knows you, you know them. They know what issues you've been facing over the years. They know how it's progressed or how it's being controlled. Whereas the locum (temporary staff) will go through the textbook you know…. try this, this, and this. I did try that quite a while ago if you look at the notes, go back and back and back, and they haven't got time to be doing that.” (62yr, male)	H
4.23	“This leaflet, there's just broad headings. It was given to me the last time I was discharged (from endoscopy department). It's not exactly a big document. It's good, I know now what Barrett's is. But so what? If something leaves the question of ‘so what?’, it hasn't done enough.” (70yr, male)	L
	“I've had very little information from health professionals. I've had to educate myself with Dr. Google which is not brilliant.…no dietary or lifestyle advice whatsoever. Again, it was down to me to search that out.” (66yr, male)	F
4.31	“I would have liked to know what caused it. What are the chances of it, you know, becoming cancerous? What treatment is available? I would have just liked to know more about it really. It's a bit scary.” (61yr, female)	K
4.32	“I'd like someone with knowledge to be able to talk me through it, the pros and cons, the risks, and what the standards or whatever they would be, to be applied but with knowledge, not just to be given the briefest bit of information but given options as well.” (66yr, male)	F
	“I don't think my doctor (referring to GP) would be able to give the right level of reassurance because they're not going to have that day‐to‐day practise of working in that area.” (46yr, male)	J
	“Whoever's on duty at the time, obviously know about Barrett's, but obviously don't have a big interest in it. Like I said when you're going in (e.g. to an endoscopy appointment), everybody's going for something different aren't they. When I was going in they said…. ‘What are you coming in for?’. If it was a specialist they would know what I was coming in for, wouldn't they.” (66yr, male)	P
	“If you're speaking to someone specialising in it, that's their main interest, so you've got their attention. Plus, you know, there's always someone there who understands the condition and if you have got any concerns you feel like they know what you're talking about.” (63yr, male)	S
4.33	“I think the mannerism with the staff helps an awful lot. When you walk into an atmosphere where everybody is pleasant type of thing that helps settle you down. If the people who are doing it are anxious that would make you more anxious. And it's always nice to know that the people around you know exactly what they're doing.” (58yr, male)	G
4.44	(re dedicated clinic) ““ I think that's what is really needed to be quite honest, from my point of view. There's just not enough information out there, concrete information. I think it gives more confidence to the patient, rather than just saying “look at this information leaflet and follow that to the best you can.”” (66yr, male)	P
	(re dedicated list) “That would be good because, obviously, the man with the camera is just doing one after another probably different procedures, like I said he's no specialist in Barrett's. I mean when you're going in, they had to ask me what I am coming in for. I think it will be a lot better. Obviously, if they're more trained in Barrett's they know what they're looking for.” (58yr, female)	R
	(re nurse care provider) “that would be good as long as they specialise in that particular area. Because for example when you ask your GP, sometimes he won't want to commit or wrongly advice you, and sometimes he'll probably just look on Google. (42yr, male)	D
	(re nurse/doctor care provider) “I don't think it makes any difference as long as they are keyed up on the subject, why should it?” (58yr, male)	G
	(re online clinic) “Well, it goes back to banking doctor, my husband and I are old school we like to speak to somebody at the bank over the counter because we're not into the internet. It's nice to speak to somebody.” (76yr, female)	Q
	(re online clinic) “I mean people's IT skills are improving all the time, and mine are okay, but I still don't think it's the most appropriate way to deal with things because it's impersonal.” (62yr, male)	H

### 4.1 Burden of disease

#### 4.1.1 Importance of symptom control

All patients reported effective long‐term symptom control due to the positive impact of medication and/or lifestyle interventions with little impact on their activities of daily living. Achieving consistent symptom control remains highly important to patients as most recall a significant impact on their quality of life before treatment. Some also report disruptive symptom flare‐ups which interfere acutely with their quality of life, for example social occasions. These can be unpredictable and challenging to manage. The strategies adopted, confidence and ability of patients to self‐manage flare‐ups vary widely. Active symptoms also appear to cause anxieties regarding disease progression and worry of oesophageal cancer with some participants seeking medical attention and sooner endoscopies via their GP.

#### 4.1.2 Worry and anxiety of oesophageal cancer

Some participants are able to put thoughts regarding cancer “to the back of their mind” or approach cancer risk pragmatically with a “what will be will be” attitude. One participant's perspective of BO cancer risk changed dramatically to one of little significance after receiving a diagnosis of a more life‐threatening disease (F, 66yr, male). However, many patients do report worry or anxiety regarding developing oesophageal cancer. This appears to be most strongly associated with times of poor symptom control or in the weeks preceding their surveillance endoscopy. There was no correlation with degrees of cancer worry and participants’ length of BO (Prague classification), a recognized individual risk factor. Factors that seem to enhance or precipitate worry include an anxious pre‐disposition, past or personal experiences of cancer, having dependants, inaccurate or poor disease‐specific knowledge and waiting times on the day of their surveillance test or indeed in the weeks afterwards for biopsy results.

Participants with more adequate disease‐specific knowledge and an internal locus of control seemed to report less cancer‐related worry. Immediate verbal communication of surveillance test results also helped prevent anxiety over biopsy results in the weeks following endoscopy. Enrolment into surveillance was also a big factor in helping reduce worry of cancer. Considering the lack of RCT evidence for the efficacy of surveillance, nearly all participants, perhaps wrongly, overvalue its protective effects. When asked about their response to an overdue surveillance test (i.e. exceeding the planned or expected surveillance interval), nearly all participants would actively chase this up and would strongly question health professional advice to discontinue surveillance.

#### 4.1.3 Burden of surveillance endoscopy

Anxiety and worry surrounding surveillance endoscopy are not solely related to thoughts of disease progression but to the physical implications of the test. Many patients find the test physically burdensome, intrusive with a sense of it being out of their control. The main physical distresses reported were during the test rather than afterwards, and these included difficulties swallowing the camera, uncomfortable retching, choking and coughing. In such cases, anxieties can build from the moment they receive the appointment and climax on the day of the procedure, and this is exacerbated further by the waiting time in endoscopy. Effective communication from health‐care professionals in the procedure room appears vitally important in counteracting this and helping them cope.

### 4.2 Follow‐up experiences

#### 4.2.1 Inadequate follow‐up at diagnosis

Participants’ experiences of secondary care follow‐up at the time of their diagnosis were inconsistent and in the majority of cases inadequate for their needs. The majority of patients received a brief interaction post‐endoscopy either from the endoscopist or from the nurse at discharge. In some instances, BO was not discussed at all. In these cases, participants received notification via a copy of their endoscopy report or subsequent letter. In one case, the participant was unaware of the diagnosis until they were asked to attend for their next surveillance endoscopy. Such inconsistencies and inadequacies could be predicted considering the BSG has only recommended outpatient clinic follow‐up since their latest guideline publication in October 2013. However, those who did receive clinic follow‐up also reported mixed experiences with some feeling the clinic was too time‐pressured, with a lack of emphasis on Barrett's and left with unanswered questions.

#### 4.2.2 Primary care experiences

Engagement with primary care was minimal at the time of diagnosis in most cases. Participants would, and in some cases, have relied upon their GP as the first port of call during an unmanageable flare‐up of symptoms. Those with greater continuity of care and longer‐term relationships with their GP appeared to have more satisfaction and trust in their GP's abilities to deal with their BO. However, many reported difficulties getting appointments quickly and poor continuity of care with surgeries increasingly using temporary staff. Some participants felt their GP was dismissive or lacked knowledge regarding BO with a heavier focus on medication changes rather than on lifestyle interventions.

#### 4.2.3 Inadequate disease specific information

Inadequacies of follow‐up care provisions appear to have led to poor disease‐specific knowledge in most cases with no clear association with any of the demographics collected. For example, some participants hold inaccurate views of exactly what BO is, while others over‐ or underestimate their cancer risk. Misleading or inadequate knowledge, in some cases, appears to have detrimental effects such as enhancing cancer worry or reduce their ability to self‐manage symptom flares. The majority of participants have acquired information verbally on an ad hoc basis from their GP or health‐care professionals at the time of their endoscopy with some cases receiving written information in the form of a leaflet or copy of their endoscopy report. Any written information appears welcomed by participants but this often led to further questions or, in the case of the endoscopy report, was difficult to interpret due to the use of medical jargon.

Nearly all have sought further information and are predominantly self‐educated via the Internet, newspaper articles, books or radio shows, for example. The Internet was by far the most common resource used; however, participants expressed concerns and fears over obtaining inaccurate worrisome information with no clear guidance on where to find trusted sources online. This finding was present in both younger and older participants. Some patients expressed concerns that improved disease‐specific knowledge may heighten anxieties regarding oesophageal cancer and were least likely to seek additional information preferring to adopt an “ignorance is bliss” approach. In comparison, overestimators of cancer risk were linked to heightened anxieties and worries of cancer, whereas those who correctly viewed their risk as low, generally, appeared to have less worry.

### 4.3 Follow‐up needs

#### 4.3.1 Greater disease specific knowledge

The major unmet need identified was disease‐specific knowledge, particularly at the time of diagnosis. This was apparent in those with short and long disease duration. Some patients still harbour significant unanswered questions years after diagnosis. Nearly all patients ideally would have preferred a face‐to‐face consultation after diagnosis to allow questions and, if necessary, attendance of their next of kin. Few participants would have preferred the delivery of this information via consultation immediately after their initial diagnostic procedure. Practically, this approach is less feasible when one considers sedated patients, the processing of biopsies and time pressures in an endoscopy department. Participants were able to identify current knowledge gaps and key uncertainties they would want addressing at the time of diagnosis (Table 4). Although those who received copies of their endoscopy report did not find them very informative, they did find the associated diagrams and pictures of their oesophagus both useful and interesting.

**Table 4:** Disease‐specific knowledge; patient uncertainties

Subtopic	Patient uncertainties
1. Barrett's oesophagus	What is Barrett's oesophagus?
	What causes Barrett's oesophagus?
2. Oesophageal cancer risk	What are the stages of the disease?
	What is my risk of oesophageal cancer?
3. Role of surveillance	What are you looking for during surveillance?
	Are there other options to surveillance?
4. Medical treatment	Why do I need to take PPIs long term?
	Are PPIs safe to take long term?
	Can Barrett's oesophagus be reversed?
	If things change what treatment is there?
5. Lifestyle	What can I do to improve my symptoms?
	What can I do to reduce my risk of cancer?
6. Managing acute symptoms	How can I manage symptom flare‐ups?
	When should I seek medical help?

#### 4.3.2 Value of seeing a specialist

When asked about improving delivery of care and reflecting on their past experiences, it was clear that patients highly value face‐to‐face interaction with a specialist. This probably reflects past inadequacies of secondary care follow‐up and the concerns some have over their GPs’ knowledge, ability and attitude towards BO. Potential benefits identified included greater expertise, experience, continuity of care and reassurance. Some patients also report to be more likely to follow verbal advice from a specialist than written information. Those with additional chronic health conditions, such as heart disease (T, 65yr, male), reflected warmly on other specialist input and appeared to seek the same in their BO care.

#### 4.3.3 Improved communication, organization and structure during secondary care follow‐up

Endoscopy staff (endoscopist, endoscopy nurse and health‐care worker) communication appears vital in maximizing the patients’ experience during surveillance endoscopy, in particular reassurance during the procedure and clear verbalization of encouraging endoscopy findings afterwards. Participants who had experienced endoscopy at both sites of this hospital favoured the diagnostic outpatient endoscopy suite over the acute hospital site. This was predominantly due to staff attitude, atmosphere, accessibility and waiting times in the department. Participants also sought greater continuity and fluency of care during their follow‐up. In particular, some faced difficulties when making endoscopy appointments, including chasing overdue tests.

#### 4.3.4 Perceptions of new models of follow‐up care

- Dedicated Barrett's oesophagus clinic and endoscopy

Patients were asked about their views on the implementation of a dedicated Barrett's service. This service, run by a health‐care professional (gastroenterologist or nurse specialist) with a specialist interest, would encompass both surveillance endoscopy and an outpatient clinic. All participants responded positively to this concept. In particular, they liked the face‐to‐face contact with a specialist and thought it could potentially solve the continuity of care issues currently faced. When asked specifically about the provider of this care, the majority of patients would be happy to see either a specialist doctor or nurse. Very few, but typically older male participants, had some reservations regarding this such as appropriate training or supervision of the nurse specialist. Individuals with other chronic diseases, such as rheumatoid arthritis, related to positive experiences with other nurse specialists (e.g. participant Q, 76yr female). Some patients eluded to other potential enhanced outcomes such as improved disease‐specific knowledge and greater reassurance. Others were surprised that surveillance endoscopies were conducted by so many different people and suggested the test may be conducted more thoroughly if done by fewer, more experienced individuals.

- Patient‐Initiated consultation

All participants were asked about their ideas, concerns and potential usage of a patient‐initiated consultation service. They were asked to consider two different approaches, firstly a telephone direct access line where patients can leave a message and be contacted back by a member of the dedicated Barrett's service and secondly, an online “virtual clinic” where patients can upload their concerns or symptoms and be contacted back in the same manner. All participants liked the overall concept of a patient‐initiated consultation, especially the direct and quicker access to specialist services which bypass and therefore free up GP time. Patients liked the idea of a reassuring “safety net” and drew comparison with other specialities, such as ENT and rheumatology, where they had benefited from similar systems. Nearly all participants preferred the telephone consultation over an online clinic. The main reason for this was the impersonal nature of using a computer and concerns over IT literacy and computer access in older generations. Some were also concerned, in general, about inappropriate use and cost of the service, suggesting there needed to be clearly defined triggers to guide self‐referral.

## 5 DISCUSSION

This study aimed to explore BO from the patients’ viewpoint, in particular the impact on health‐related quality of life, experiences and effectiveness of follow‐up care and opinion on new follow‐up systems. To our knowledge, this represents the most in‐depth account of BO patients’ perspective of disease impact in a UK NHS setting.

The most striking finding relates to patients’ experiences of follow‐up care. Historic and current follow‐up for BO appears inconsistent and often inadequate to meet patients’ needs and expectations. This has led to poorly informed patients with limited or inaccurate disease‐specific knowledge. Some potential impacts, identified in this study, include reduced confidence and ability to self‐manage symptoms and heightened cancer‐specific worry. Very few studies exist which assess levels of patient education in BO. Concerningly, in 2008, Murphy and colleagues reported <50% of patients with concurrent OAC and BO diagnoses were aware of their BO diagnosis despite an average of more than seven previous endoscopies.^32^ Improved patient knowledge in IBD appears to have positive and detrimental effects with greater knowledge associated with adaptive coping strategies but also higher anxiety levels.^33^, ^34^ This reflects those who report an “ignorance is bliss” attitude too improving disease‐specific knowledge in this study. Even participants with longer‐term diagnoses voiced unmet needs and questions regarding their condition. This finding questions the current BSG guidance which only recommends new patients attend an outpatient clinic.^5^ The role of a Barrett's clinic may be much broader than this, by giving all patients the option of attending clinic after their surveillance endoscopy would capture patients seeking more information and guidance about their condition. In some cases, it may also provide a platform for addressing poor symptom control or an opportunity to discuss the appropriateness of discontinuing surveillance. The latter may be vital when one considers the number of patients who may have been enrolled in surveillance inappropriately^35^, ^36^ or indeed in a time when diagnostic criteria were less clear. Discussions regarding cessation of surveillance are unlikely to be adequate or satisfactory to patients at the time of endoscopy as this study has shown patients hold strong beliefs regarding its protective efficacy. A clinic appointment specifically to explain the reasons for cessation of surveillance, for example in medically unfit patients where the risks outweigh the benefits, may help patients understand and accept the physicians’ recommendation with less anxiety.

The findings suggest that BO patients have three key potential impacts on their HRQOL: symptom control, worry of oesophageal cancer and burden of surveillance endoscopy. Overall patients generally report good long‐term symptom control with little impact on their daily lives. This finding may reflect previous quantitative work which shows reflux symptoms in BO cohorts are commonly better than those with a diagnosis of gastro‐oesophageal reflux disease.^37^, ^38^, ^39^ However, consistent control remains imperative as a significant minority suffer from symptom flare‐ups which interfere with activities of daily living and, in some cases, trigger worries of disease progression, specifically oesophageal cancer. This intermittent effect may not be captured during quantitative HRQOL questionnaire assessments when one considers the lack of a validated BO patient reported outcome measure and varying questionnaire recall periods.

The other chief trigger of cancer worry is an approaching surveillance endoscopy. This acute worry may be harder to modify and is a well‐documented impact of cancer prevention activities.^40^ Pretest worry and anxiety were also strongly associated with the physical implications of the test with many patients reporting the endoscopy as physically burdensome. Although past research suggests patients undertaking the test for symptoms rather than BO surveillance find the test even worse, implying patients’ burden may reduce with repeated exposure.^41^ Enhancing patients’ experience of surveillance endoscopy appears multifactorial but should focus strongly on health‐care professional communication during and after the procedure. These findings are supported by previous quantitative work. Kruijshaar and colleagues reported lower anxiety scores after endoscopy than beforehand in BO patients.^41^, ^42^ This probably reflects reprieve from reassuring results and the relief of completing a physically taxing test. However, anxiety levels in this study remained raised one month after endoscopy when compared to those who underwent endoscopy for non‐specific upper gastrointestinal symptoms. This may reflect unnecessary anxiety over biopsy results or indeed a more chronic issue.

There is a clear need for change in BO follow‐up care. In particular, patients require greater information at the time of their diagnosis. This finding is comparable to research in other chronic diseases, in particular IBD where there has been development of knowledge measurement tools^43^, ^44^ and research highlighting the positive effects of a patient education.^45^ Patients clearly value the role of a face‐to‐face consultation with a knowledgeable health‐care professional. This two‐way discussion should cover both the professional (for BSG clinic agenda, see Appendix S1) and patient agendas (Table 4) with the adjunct of visual aids, ideally diagrams or pictures from their own endoscopy. Patients should also be given the option of additional written information or Website details. Patients strongly believed this should be an aide to discussion not a replacement of it. It was also clear that patients’ experiences at endoscopy varied widely with a lack of continuity of care. To improve patients’ experiences, health‐care professionals should focus on clear reassuring communication within the endoscopy room including verbalization of encouraging results to minimize post‐endoscopic worry. This finding is supported by previous qualitative work which identified factors that may influence patients’ adherence to BO surveillance. The doctor‐patient relationship was deemed vital in particular, communication prior to, interaction during and levels of trust after endoscopy.^46^ Other, logistical, areas of consideration for endoscopy departments should include waiting times on the day of procedure, ease of making appointments and the potential influence of a more calming “non‐acute” atmosphere for patients. It may be favourable for surveillance patients to attend an evening or weekend list when departments are less busy and waiting times are less likely to be lengthened by the demands of acute care. In hospital trusts with two separate endoscopy sites (elective and acute), the environment for surveillance endoscopy in the elective site is more likely to be ideal.

To develop BO follow‐up care, this study not only assembled patients’ past experiences, but sought their views on how to enhance care including their opinions on suggested new models of follow‐up. Alternative approaches to care were met positively, in particular a dedicated service which would provide a lynch pin between clinic and endoscopy at a secondary care level. This may address their main needs surrounding specialist input, improved continuity of care, organization and structure. In other studies, patient preference towards follow‐up care provider (secondary vs primary care) after cancer survival is mixed and appears influenced by multiple patient and provider factors^47^ which may vary significantly across diseases and health‐care systems. This study showed a strong patient preference towards improving secondary rather than primary care follow‐up. This likely reflects a lack of GP emphasis on BO coupled with poor continuity of care experienced. Patients are also aware this is largely an endoscopically monitored disease and therefore may lean towards a secondary care point of contact to facilitate access to endoscopy if necessary. As the BO research landscape moves forward, guidelines will change and newer surveillance endoscopy techniques are likely to be adopted. A dedicated service would also allow easier transition ensuring up‐to‐date, consistent and standardized care. In fact, some of the concerns regarding enhanced endoscopic surveillance techniques relate to their reproduction outside tertiary settings and additional training required for multiple endoscopists.^48^

Participants also liked the concept of a “safety net” in the form of a patient‐initiated consultation service. This probably reflects the potential impact of uncontrollable symptom flares, length of time in‐between endoscopies and doubts over primary care ability to deal with their concerns in a timely manner. Patients had a strong preference to a telephone‐based system rather than an “impersonal” virtual clinic which may exclude patients who lack computer access or IT literacy. This is in contrast to other chronic diseases, for example IBD, where e‐health technologies have been both acceptable and beneficial.^49^, ^50^, ^51^ This likely reflects the average age of 65 years in UK BO surveillance cohorts.^11^ It is unclear how frequent patients will engage with this service, and its wider benefits are hard to measure. Such benefits may include freeing up GP time, addressing worrisome symptoms, improving access to or preventing overuse of endoscopy. Nevertheless, this should be piloted cautiously to assess the appropriateness of use and patient satisfaction.

Based on the findings of this study, we propose the implementation of a dedicated service encompassing a Barrett's clinic, surveillance endoscopy list and direct access line. This complex care intervention could be delivered by a nurse endoscopist alongside a consultant gastroenterologist, both with a specialist interest in BO. Further research will be needed to assess the practicalities and efficacy of this intervention. Ideally, this should be prospective and randomized compared to current standard practice. Considering its complexity, there must be multiple outcome measures or a dedicated BO PROM which captures all aspects of the patients’ perspective (symptom control, worry of cancer, disease‐specific knowledge and burden of endoscopy). Once psychometrically validated, such a score would make BO HRQOL assessment less cumbersome, more sensitive and consistent, with greater allowance for cross‐study comparisons in future clinical trials. Further consideration would be needed regarding the potential clinical outcome measures, for example dysplasia diagnosis rates, which are out of the remit of this paper.

### 5.1 Strengths and limitations

The study utilized a number of steps to ensure rigour in its design; however, there are some limitations. Firstly, participants in this study were recruited through a single district general hospital population. Therefore, one must be cautious when generalizing these findings, especially those relating to organization and structure of care which may differ significantly elsewhere. However, most UK NHS hospitals provide BO care in a similar ad hoc fashion and experience the same issues organizing and budgeting follow‐up care provisions. Secondly, the study did not take a longitudinal approach to identifying BO impact over the life course. However, participants were recruited until the researchers were happy that thematic saturation was achieved with good variation of age, disease duration and gender. Variation in socio‐economic status and health literacy was not formally sought, and this may be an area for future research to clarify. Thirdly, the data captured may have been influenced by the status of the interviewer.^52^ Fourthly, only two interviews were coded by two separate researchers which may introduce bias; however, there was a strong correlation between findings and all authors reviewed and agreed upon the final themes and credibility of the analysis. Finally, all participants were “white British” and English‐speaking, so one must be cautious when translating these findings to more diverse ethnic populations. A greater number of male than female participants could be viewed as a limitation; however, this is a disease predominantly affecting men with a male/female sex ratio of 1.96/1 reported in a meta‐analysis.^53^

### 5.2 CONCLUSIONS

This qualitative research provides an in‐depth account of the patient perspective of BO in an NHS setting. Key potential impacts on patients include symptom control, worry of oesophageal cancer and burden of surveillance endoscopy. These factors must be considered when implementing future care pathways, designing clinical trials or developing a BO‐specific patient‐reported outcome measure. Follow‐up care, at this NHS hospital, was found to be inconsistent and often inadequate to meet patients’ needs. Patients require greater disease‐specific information, enhanced communication, organization and structure of care. To improve patient experiences, we recommend the design, implementation and prospective assessment of a complex care intervention, which encompasses dedicated BO surveillance, outpatient clinic and telephone direct access line.

## CONFLICT OF INTEREST

Nothing to disclose.

## AUTHOR CONTRIBUTIONS

All authors significantly contributed to this work. The concept and design of this study was instigated by Professor Yeng Ang, Professor John McLaughlin and Professor Shaheen Hamdy. The interviews were conducted by Dr James Britton. Associate Professor Maria Horne and Dr James Britton conducted the analysis and interpretation of the interviews. Dr James Britton drafted the initial manuscript. All authors then had a role in writing and revision of the manuscript prior to submission.
