# Supplementary material for: Barrett's oesophagus: A qualitative study of patient burden, care delivery experience and follow‐up needs
Source: Health Expect. 2018 Nov 14;22(1):21–33. doi: 10.1111/hex.12817 (PMC6351418; doi:10.1111/hex.12817)
Supplement: Supplementary file 1 [file HEX-22-21-s001.docx]

Appendix

**Table: Consolidated Criteria for Reporting Qualitative studies (COREQ) 32-item checklist**

| **No. Item** | **Description** | **Reported in section** |
| --- | --- | --- |
| **Domain 1: Research team and reﬂexivity** | | |
| *Personal Characteristics* |  |  |
| 1. Interviewer/facilitator | Interviews were conducted by JB | 2.3 Data collection |
| 2. Credentials | JB; Post graduate (MBChB) PhD student.  SH; Professor of Neurogastroenterology (non- Barrett’s specialist) and honorary consultant Gastroenterologist.  JM; Professor of Gastroenterology and Nutrition (non-Barrett’s specialist) and honorary consultant Gastroenterologist.  MH; Associate professor. Main research expertise in qualitative and mixed methods research. Clinical background in primary care, community and public health nursing.  YA; Professor in Gastroenterology (oesophageal diseases) and Consultant Gastroenterologist and Honorary reader with specialist interest in Barrett’s oesophagus. |  |
| 3. Occupation | As listed above |  |
| 4. Gender | JB; Male  SH; Male  JM; Male  MH; Female  YA; Male |  |
| 5. Experience and training | JB Training in Qualitative Research includes:  - Manchester University Tutorials  - SRA Qualitative data analysis  - Guidance from academic supervisors/senior authors |  |
| *Relationship with participants* |  |  |
| 6. Relationship established | Prior to recruitment participants and researcher had not met. | 2.2 Participants and setting |
| 7. Participant knowledge of the interviewer | Participants were made aware that JB was a doctor doing post graduate research. | 2.3 Data collection  5.1 Strengths and Limitations |
| 8. Interviewer characteristics | Participants were made aware of the interviewer’s characteristics (doctor and researcher). The potential impact of this on the data collected has been reflected upon. All authors backgrounds have been included in this checklist and the manuscript includes a COI statement. | 5.1 Strength and Limitations  Conflict of Interest Statement |
| **Domain 2: study design** | | |
| Theoretical framework |  |  |
| 9. Methodological orientation and Theory | Framework analysis approach | 2.0 Methods |
| Participant selection | Patients undergoing BO surveillance were recruited to achieve maximum variation in terms of age, gender, sex and disease duration | Table 2 |
| 10. Sampling | Purposive sampling | 2.2 participants and setting |
| 11. Method of approach | Face-to-face, telephone or mail. | 2.2 participants and setting |
| 12. Sample size | 20 | 4.0 Results |
| 13. Non-participation | 20 Participants  45 Declined to participate (reasons not explored)  0 Dropped out |  |
| Setting |  |  |
| 14. Setting of data collection | At the workplace in a private seminar room | 2.3 Data Collection |
| 15. Presence of non-participants | None | NA |
| 16. Description of sample | Participant demographics and characteristics reported include; Age, gender, disease duration, Prague classification and co-morbidities. | Table 2 |
| Data collection |  |  |
| 17. Interview guide | The topic guide was devised from prior literature review and expert opinion. | 2.3 Data collection  Appendices |
| 18. Repeat interviews | None | NA |
| 19. Audio/visual recording | All interviews were audio recorded and transcribed verbatim. | 2.3 Data Collection |
| 20. Field notes | Field notes were made during the interview. | 2.3 Data Collection |
| 21. Interview duration | Average= 40 mins  Range= 21-76 mins | 2.3 Data Collection |
| 22. Data saturation | Data saturation occurred at 20 interviews | 4.0 Results |
| 23. Transcripts returned | Transcripts were not returned to participants for comment/correction | NA |
| **Domain 3: analysis and ﬁndings** | | |
| Data analysis |  |  |
| 24. Number of data coders | 2 authors (JB and MH) | 3.1 Rigor |
| 25. Description of the coding tree | Table 1 outlines the conceptual framework (main themes and initial categories) with the number of contributing participants and verbatim quotes linked to each item. | Table 1 |
| 26. Derivation of themes | The initial themes, which formulated the conceptual framework, were derived from the first 4 interviews and topic guide. | 3.0 Analysis |
| 27. Software | NVivo Pro 11 | 3.0 Analysis |
| 28. Participant checking | No participant feedback on the ﬁndings | NA |
| Reporting |  |  |
| 29. Quotations presented | Participant quotations are presented in the supplementary material (appendix). Each quotation is linked to a unique participant ID | Appendix |
| 30. Data and ﬁndings consistent | There was consistency between the data presented and the ﬁndings. | 4.0 Results |
| 31. Clarity of major themes | Major themes are clearly presented | 4.0 Results  5.2 Conclusions |
| 32. Clarity of minor themes | Diverse findings/cases are included in throughout the results section | 4.0 Results |

**Topic Guide**

Introduction

- Explain no wright/wrong answers and the need to record the discussion
- Discuss confidentiality
- Explain this research forms part of a larger study exploring the impact Barrett’s Oesophagus and its care pathways on patients.

Objectives

- Develop a greater understanding of the patient’s viewpoint in relation to their diagnosis and care.
- Explore what factors related to Barrett’s Oesophagus affect patients Quality of life.
- Identify any particular problems patients with Barrett’s Oesophagus may experience.
- Identify any particular problems patients experience with their follow up care
- Help us explore ways of improving their follow up care.
- Identify what is important to them in their follow up and how they would change their care.

Background Information

- Participant introduces themselves (sound check)
- Include age, gender, co-morbidities and disease duration.

**Exploring the Patient Burden of Barrett’s Oesophagus**

Summary of Topics/Discussion prompts.

1. **Impact on General Health.** Explore any negative or positive issues raised. Probe into why they have these beliefs or feelings. How have these experiences affected them? How have they coped with any negative experiences?
   1. How do you consider your current general health? What concerns, if any, do you have?
   2. How has your health changed since you have been diagnosed with Barrett’s Oesophagus?
2. **The impact of Physical Symptoms.** Explore any impact on activities of daily living including work, leisure, sleep, relationships etc.). Probe into how these symptoms may have affected them and how have they coped with any negative experiences?
   1. How do you control your symptoms related to Barrett’s Oesophagus (i.e. GORD)?
   2. In what ways have you had to change your lifestyle? (eating habits, smoking, alcohol)
   3. Do you ever have any problems or concerns regarding the medication you take for Barrett’s Oesophagus?
   4. What typically happens when you experience breakthrough/uncontrolled symptoms?
   5. How important is adequate symptom control to you? (may need to ask patients to think back to pre-diagnosis/pre-medication)
3. **The Psychological Burden.** Explore any impact on activities of daily living including work, leisure, sleep, relationships etc.). Probe into why they have these beliefs or feelings and how these experiences may have affected them. How have they coped with any negative experiences?
   1. What concerns or worries do you have relating to Barrett’s Oesophagus?
   2. How do you perceive the risk of developing Oesophageal cancer in people with Barrett’s Oesophagus?
   3. How do you perceive your own risk of developing Oesophageal cancer?
   4. How else has a diagnosis of Barrett’s Oesophagus affected your mental health?
4. **The Burden of Endoscopic Surveillance?** Explore any positive and negative issues raised. Probe into why they have these beliefs or feeling and how these experiences may have affected them. How have they coped with any negative experiences?
   1. What concerns, if any, do you have regarding the need for repeated endoscopies?
   2. What aspect/s do you find the most burdensome? (Explore before, during and after the test)
   3. How important are surveillance endoscopies to you?
   4. If your next surveillance endoscopy was missed or late how would this affect you?
   5. How would you feel if someone said you no longer required endoscopy check-ups?

**Exploring the follow up needs of patients with Barrett’s Oesophagus**

Summary of Topics/Discussion Prompts

1. **Experience of follow up care at diagnosis**. Explore positive and negative issues and how these experiences have impacted them. Probe into why they have these beliefs or feelings. How have they coped with any negative experiences raised?
2. What occurred at the time of initial diagnosis? Probe: What concerns, if any, did you have and explain further…
3. How were you followed up?
4. What did you want or need at this time?
5. How did you receive information about your diagnosis and future surveillance? (at endoscopy, clinic, leaflet, self-educated)
6. Did they engage with their GP for support or advice? Explore any barriers.
7. Probe: Who provided this information? Did you feel the information provided was adequate? If not, why not? Explain further?
8. Overall was this follow up adequate enough? Were you provided/equipped with everything you needed regarding Barrett’ Oesophagus? (such as knowledge, medication, lifestyle advice, symptom control etc).
9. **Experience of follow up care now**. Explore positive and negative issues and how these experiences impacted them. Probe into why they have these beliefs or feelings. How have they coped with any negative experiences raised?
   1. Typically, what happens with your current follow up?
   2. How important is follow up care to you now?
   3. How have your needs changed from diagnosis to now?
   4. How important is the “doctor-patient” face to face relationship? Explore both primary/secondary care.
   5. How satisfied are you with your current follow up arrangement?
10. **Changes to current follow up care**. Explore any knowledge of alternative methods. (If no knowledge then describe alternatives to generate discussion e.g. direct access line, virtual clinics, open access patient initiated appointments etc.)
    1. If you developed breakthrough symptoms or concerns regarding your Barrett’s Oesophagus how would you manage this?
    2. At what point would you ask for help and who would you contact?
    3. Have you considered any other means of follow up?
    4. What do you think about a specialist Barrett’s Oesophagus service? (clinic and endoscopy)
    5. What do you think about patient-initiated appointments?
    6. What do you think about telephone consultations or online remote “virtual” clinics?
    7. What would you change about the current follow up system? (Explore where, how, by whom and why)

**Summary Question: Are we missing anything.** Is there anything else that I have failed to ask you in this interview which you feel is important for me to know?

Table: Example of a thematic chart (disease impact)

|  | **Sub-Themes** | | |
| --- | --- | --- | --- |
| **Responder** | **2.1 Physical symptom impact** | **2.2 Associated worries or anxieties** | **2.3 Surveillance endoscopy impact** |
| **A** | “I thought I’m not putting up with it because…..I sing in a choir and I felt it was affecting me” |  |  |
| **B** |  | “I don’t worry about it. What will be, will be. And if it’s not that bad I’m not going to worry about it” |  |
| **C** |  |  | “once or twice when the camera twists it makes you gurgle, it’s like you’re choking” |
| D | “I struggle when say you’re going out with friends and it comes on, it’s hard to explain, you can’t go around and tell them, you just need to go home and you just feel like you want to be sick.” |  |  |

Table: BSG Guideline for the Barrett’s oesophagus clinic agenda

| **BSG Guidance** | **Barrett’s Oesophagus Clinic Agenda Topics** |
| --- | --- |
| Logistics | Patients should receive an early outpatient appointment (ideally within 4–6 weeks)  With a physician with a clinical interest in Barrett’s |
| Discussion | - Diagnostic Certainty   - Whether or not there is an indication for endoscopic surveillance (Considerations include the likelihood of cancer progression, patient fitness for repeat endoscopies and patient preference)   - If there is need for further diagnostic work up this should be clearly explained to avoid confusion - Cancer Risk; low but significant cancer risk. - Possible lifestyle changes - Surveillance Advantages; The possible benefits of surveillance in detecting early-stage tumours and improving cancer survival - Surveillance Disadvantages   - Should also mention the lack of randomised controlled data to prove the benefits of surveillance   - Clinicians must emphasise to the patient that the actual risk of death from oesophageal cancer is small   - Disadvantages of endoscopy surveillance should also be discussed, including the small risks of the procedure and the associated psychological morbidity   - Failure rate in that surveillance cannot guarantee to detect every tumour that may develop - Future Potential therapeutic options if dysplasia is detected (endoscopic and surgical) |
| Documentation | - Family history for Barrett’s oesophagus and OAC should also be recorded |
| Written Information | - BSG Online supplementary appendix 4 - Other approved materials such as MacMillan, CancerBACUP |
| Assessment | - Patients should be fit for repeated endoscopy procedures and endoscopic therapy if HGD or early cancer is detected - Consider endoscopic surveillance in patients with PS 0–2, provided that the estimated patient life expectancy is sufficiently long for the individual to benefit from surveillance if dysplasia or early cancer were detected. |
